# Psychiatric burden in a cohort of adults with Niemann Pick type C disease: from psychotic symptoms to frontal lobe behavioral disorders

**DOI:** 10.1186/s13023-023-02851-x

**Published:** 2023-09-22

**Authors:** A. Morin, G. Carle, A. Ponchel, G. Fernández-Eulate, Y. Nadjar

**Affiliations:** 1 Department of Neurology, Rouen University Hospital, University of Rouen, 76000 Rouen, France; 2 Department of Psychiatry, Rouvray Hospital, University of Rouen, 76000 Rouen, France; 3 Saint-Exupery Private Clinic, Toulouse, France; 4 GHU Paris Psychiatrie & Neurosciences, Paris, France; 5 Neuro-Metabolism Unit, Neurology Department, Reference Center for Lysosomal Diseases, Pitié-Salpêtrière University Hospital, APHP, Paris, France; 6 Institut Necker-Enfants Malades, INSERM U1151, BioSPC (ED562), Université Paris Cité, Paris, France

**Keywords:** Psychosis, Neuropsychiatry, Niemann-Pick type C, Lysosomal storage disorders, Behavioral disorders

## Abstract

**Objectives:**

To describe Niemann-Pick type C (NP-C) behavioral symptoms (focusing on psychotic symptoms) and its relation to frontal lobe functioning.

**Methods:**

We retrospectively reviewed medical charts of NP-C-patients followed in the Lysosomal Diseases reference center in Paris Pitié-Salpêtrière. We collected demographic data, psychiatric clinical manifestations, psychometric scales, and extended neuropsychological data including executive and behavioral frontal lobe functions evaluations.

**Results:**

Nineteen patients were included in the study with ten of them having experienced at least one acute psychotic episode, being inaugural for six of them. Most of the patients suffered from behavioral (15/17) and cognitive disorders (18/19) (including executive dysfunction (11/12), apathy (13/17), impaired social cognition (11/13) and stereotyped behaviors (5/10). For five patients, quality of life was significantly impaired by these abnormal behaviors. Concerning frontal neuropsychological evaluation, Facial emotion recognition was by far the most performed neuropsychological test (n = 8) and the score was always abnormal. It is noteworthy that psychotic symptoms were often drug resistant (8/9) and that Miglustat was associated with a better control of psychotic symptoms.

**Conclusions:**

We report a high frequency of psychiatric symptoms in NP-C encompassing acute psychotic manifestations, often presenting early in the course of the disease with atypical features. We also report disabling behavioral manifestations related to frontal dysfunction.

**Supplementary Information:**

The online version contains supplementary material available at 10.1186/s13023-023-02851-x.

## Introduction

Niemann-Pick type C (NP-C) is a lysosomal storage disorder (LSD) resulting from bi-allelic mutations in *NP-C1* or *NP-C2* gene, with clinical manifestations that may begin from perinatal to adult life [27]. *NP-C1* and *NP-C2* genes code for *NP-C1* and *NP-C2* proteins, localized respectively in the lysosome membrane and lumen, functioning together for cholesterol export from the lysosome, and leading when altered to subsequent cholesterol lysosomal accumulation, but also ganglioside secondary lysosomal accumulation especially in the nervous system. *NP-C1* is involved in 95% of patients, whereas *NP-C2* is involved in 5%, with reported more severe disease and exceptional adult onset forms. The prognosis of the disease, apart from the potentially fatal visceral neonatal form, is clearly related to the age of neurological onset, that defines the different forms of NP-C (ie early infantile, late infantile, juvenile, or adolescent/adult). Children with NP-C, especially younger patients, may experience a very severe neurological disease with a psychomotor regression leading to death after a few years, associated with overt systemic disease (splenomegaly, hepatomegaly, sometimes lung disease), whereas adults have a more protracted course with survival possible along several decades. In addition to prognosis, clinical picture is clearly different in adults: NP-C mainly presents as a neuropsychiatric degenerative disease, that associates motor (cerebellar ataxia, dystonia, myoclonus), cognitive and psychiatric manifestations with no or little systemic involvement [26]. Blood diagnostic biomarkers are now currently used for NP-C screening, with high sensitivity and specificity [22, 30]. Achieving an early diagnosis is crucial since patients can benefit from miglustat treatment, a substrate reduction therapy that has shown efficacy in stabilizing or slowing the disease progression, particularly in adults when neurological disability is not yet severe (ie, before the onset of dementia, loss of ambulation, or need of gastrostomy feeding) [26, 29]. However, first NP-C manifestations are often non-specific, delaying diagnosis and treatment introduction.

Psychiatric manifestations in NP-C disease have been reported for more than two decades now, especially in the form of acute psychotic episodes, often as the initial manifestation of the disease [9, 26, 29]. However, as diagnosis of NP-C is usually achieved several years later, clues about the semiological key points of the first acute psychotic episode are scarce. There is nonetheless a need to rationally identify those patients who could benefit from a blood NP-C screening among the numerous ones presenting with an acute psychotic episode of unknown origin. Beyond psychosis, behavioral symptoms are less often reported, however we observed that these could represent a major burden in some NP-C patients, even more than motor and cognitive impairment [9]. Therefore, we aimed to study the specific characteristics of behavioral manifestations in NP-C, during the first acute psychotic episode and beyond. For this, we report here detailed retrospective data from medical charts, including semi-structured interviews, psychometric scales and neuropsychological evaluations from a single-center French cohort of adult patients with NP-C.

## Material and methods

### Participants

This retrospective study was performed from 2016 to 2020 in the department of Neurology of Pitié-Salpêtrière hospital in Paris, France, hosting the Lysosomal Diseases Reference Center and a behavioral neuropsychiatric ward.

Patients included were seen in our center between 2016 and 2020, or were identified in the Lysosomal Diseases Reference Center database as NP-C patients who experienced a psychotic episode (30 NP-C patients in the database in 2020, all referred to our reference center). All included patients had a genetically confirmed NP-C disease (two mutations). Data from medical charts were reviewed and collected for all patients. This included demographical data, key items concerning NP-C disease (diagnosis, clinical features, evolution), description of the psychiatric manifestations, psychometric scales data and neuropsychological evaluation data. Notably, psychiatric manifestations were collected from psychiatric reports in different French centers involved along the whole psychiatric history of the patients, and from semi-structured interviews (patients and/or parents) performed in the behavioral neuropsychiatric ward in Pitié-Salpêtrière Hospital (during consultations or phone calls). Psychotropic drugs were listed, including all classes of psychotropic medication. Absence of Anti-psychotic (AP) efficacy was defined as the persistence of symptoms despite more than 2 trials of adequate dose and duration of antipsychotic medication with documented adherence. Psychotropic drug intolerance was defined by the occurrence of well-known side effects related to the specific psychotropic medication prescribed to the patient and reported in the patient’s medical records.

### Statistical analysis

Data analysis was performed using SPSS 26 (IBM). Concerning continuous values: Mean and median values, standard deviation, and range were reported using SPSS data description function. Concerning categorical values: absolute or relative frequencies of categorical variables were reported using SPSS data description function.

### Ethical statement

This study was conducted according to French legislation and authorized by the National Commission on Informatics and Liberty (no. 2211991). The patients were informed about the utilization of their anonymized data in this study.

## Results

Nineteen patients with NP-C followed in our center were included in the study (see demographic and NP-C related data in Table 1), with ten of them having experienced at least one acute psychotic episode. Due to missing data, we report the ratio of patients in whom a symptom was reported out of all the patients for whom the data was available. Whereas all patients were considered as adult-onset NP-C (onset above 10 years-old), we observed subtle previous manifestations during childhood before onset of overt disease in 10/18 patients, including psychomotor delay in 6/17, learning disorder in 6/17, and autistic symptoms in 3/13.

**Table 1:** General characteristics and psychiatric manifestations in 19 adult NP-C patients

Disease characteristics							Development		
Patient ID	Gender	Age at first manifestations	First manifestations	Psychosis	Age at diagnosis	Age at last follow-up	Psychomotor delay	Learning disorder	Autistic symptoms in childhood
1	F	10	Ataxia, dysarthria, dysphagia	+	18	36^a^	+	−	NA
2	F	12	VSGP, ataxia	+	22	34^a^	+	−	+
3	M	15	Ataxia	+	23	32	+	−	NA
4	M	16	Psychosis, cognitive	+	32	38	NA	NA	−
5	M	16	Psychosis, cognitive	+	24	40	−	+	+
6	F	18	Psychosis, cognitive	+	33	50^a^	+	−	−
7	M	19	Ataxia, dysarthria, dysphagia	+	23	24	+	+	+
8	M	20	Psychosis	+	25	33	−	−	NA
9	F	25	Psychosis, ataxia, cognitive	+	27	36	−	−	−
10	F	30	Pyschosis	+	41	57^a^	−	−	NA
11	F	13	Ataxia	−	22	31	−	+	−
12	M	16	VSGP, dysarthria, cognitive	−	20	28	NA	NA	NA
13	M	16	Ataxia, dysarthria	−	23	27	−	−	−
14	F	16	VSGP	−	28	56^a^	−	+	−
15	M	18	Cognitive	−	33	35	−	−	NA
16	M	18	Deafness, cognitive and behavioural	−	35	41	−	+	−
17	M	27	Ataxia, deafness	−	36	44	−	−	−
18	M	32	Deafness, cognitive	−	33	42	+	+ ^b^	−
19	F	50	Deafness	−	65	65	−	−	−
Means or percentage	11 M/8F	20.37		52 (10/19)	29.63	36.86	35 (6/17)	35 (6/17)	23 (3/13)

Thymic symptoms		Behavioural symptoms								Cognitive disorder	Treatment
Depressive symptoms	Anxiety symptoms	Emotional hyper reactivity	Excessive spending	Impaired social cognition	Affective blunting	Desinhibition	Social retreat	Apathy	Stereotypies		Miglustat (Mgst)
−	NA	+	−	+	+	−	+	+	NA	+	+ ^c^
+	+	+	+	+	+	+	−	−	NA	+	+
+	+	+	−	NA	−	+	+	+	NA	+	+
+	+	+	+	NA	NA	+	+	+	NA	+	+
−	NA	−	−	+	−	+	+	+	+	+	−
−	NA	+	NA	NA	NA	NA	NA	+	NA	+	−
+	+	+	+	+	+	−	+	+	+	+	+
−	−	−	+	+	+	+	+	+	+	+	+
−	NA	−	+	+	−	+	+	+	+	+	+
+	NA	+	−	+	−	+	+	+	NA	+	−
−	−	−	−	+	+	+	+	+	−	+	+
NA	NA	NA	NA	NA	NA	NA	NA	NA	NA	+	+
−	−	+	−	+	+	−	−	−	−	+	+
−	−	−	NA	−	−	−	−	−	−	+	+ ^d^
−	−	−	+	NA	+	NA	+	+	NA	+	+
+	+	+	NA	+	+	−	+	+	−	+	+
−	NA	NA	NA	NA	NA	NA	NA	NA	NA	+	+
−	−	NA	NA	+	+	+	+	+	+	+	+
−	−	−	−	−	−	−	−	−	−	−	+
33 (6/18)	42 (5/12)	56 (9/16)	46 (6/13)	84 (11/13)	84 (1/13)	60 (9/15)	75 (12/16)	76 (13/17)	50 (5/10)	95 (18/19)	84 (16/19)

^a^deceased patients

^b^intellectual disability

^c^4 years after diagnosis

^d^9 years after diagnosis

For the ten patients with an acute psychotic episode (see Table 2), this was considered as the first neuropsychiatric manifestation of the disease in six of them, even though patient 9 also suffered from hearing loss in early childhood of unknown origin (and probably related to NP-C). Globally, the first acute psychotic episode occurred at a median age of 20.5 years old (yo) (range = 16–30). In 9/10 psychotic patients, NP-C diagnosis was made after the first psychotic episode, with a median delay of 5 years (range = 0–16 years). Only patient 1 suffered from their first psychotic symptoms after NP-C diagnosis, with occurrence of isolated visual hallucinations in a sleep disorder context (diagnosed secondary narcolepsy) that both lasted a few months before spontaneous regression without anti-psychotic drug use. Delirium was frequently associated (8/10) as well as delusions (8/10) which were most frequently complex (5/8; defined as a delusion including multiple themes concomitantly), persecution-type being systematically reported. Auditory hallucinations were frequent (7/10 including four patients with no verbal hallucinations, i.e. hearing non-discriminative noises), as well as visual hallucinations (5/9). During the acute psychotic episode, behavioral symptoms classically associated with frontal dysfunction were frequently observed: emotional hyperreactivity (7/10), excessive spending (5/10), impaired social cognition (7/7), affective blunting (4/8), disinhibition (7/9), social retreat (8/9), apathy (9/10) and stereotyped behaviors (4/4). 9/10 patients with psychosis received several psychotropic drugs (median = 8 different molecules, range = 3–13), especially antipsychotic agents (median = 4, range = 1–6) as lack of efficacy was frequently reported (8/9 patients). One patient suffered from antipsychotic intolerance. Along a median follow-up of 9 years (range = 1–17) after NP-C diagnosis, 5/10 patients experienced a psychotic episode relapse, including all three patients who had never been treated with miglustat. Miglustat was associated with better control of psychotic symptoms, including decreased of antipsychotic agents need (number and/or dosage) in 5/6 patients (excluding patient 1, see above). To qualitatively illustrate semiological specificities and evolution of psychosis in NP-C, we detail the anamnesis of three patients with inaugural psychosis (patients 5, 8 and 10; see Box 1) (Additional file 1).

**Table 2:** Semiological characteristics of the psychotic episodes in 10 adult NP-C patients

Psychosis							Delusions					Other associated symptoms				
Patient ID	age at psychosis	Psychosis at onset	Age at Miglustat (Mgst)	Psychotic relapse	Age at last follow-up	Follow-up of psychotic patients after diagnosis	Types of delusions	Systematization	Visual hallucinations	Auditory hallucinations	Consciously perceived Hallucinations	Delirium	Agitation	Derealisation	Anosognosia	Discordance
1	23	−	22	−	36	NA	None	−	1	0	−	−	+	−	−	+
2	18	−	22	−	34	12	Persecution, megalomania	−	0	0	NA	+	+	+	+	+
3	23	−	23	−	32	9	Persecution, culpability	−	1	1	NA	+	+	+	+	−
4	16	+	32	−	38	6	Persecution, prejudice, culpability	−	1	1	−	+	+	−	+	+
5	16	+	No Mgst	+	40	16	Persecution	−	1	1	−	+	+	NA	+	+
6	18	+	No Mgst	+	50	17	Persecution*	−	NA	1	−	+	+	+	+	+
7	21	−	23	−	24	1	Persecution	−	1	0	−	+	+	+	+	+
8	20	+	25	+ (toxic use)	33	8	Persecution, megalomania, filiation, mystic	−	0	1	−	−	+	−	+	+
9	25	+	27	+	36	9	None	−	0	1	−	+	+	+	+	+
10	30	+	No Mgst	+	57	16	Persecution, jealousy, prejudice, culpability	−	0	1	NA	+	+	+	+	+
Means or percentage	21.00	60 (6/10)	24.86	50 (5/10)	38.00	10.44		0 (0/10)	55 (5/9)	70 (7/10)	0 (0/7)	80 (8/10)	100 (10/10)	66 (6/9)	90 (9/10)	90 (9/10)

Associated thymic symptoms		Associated behavoural symptoms								Psychotropic treatments			
Depressive symptoms	Anxiety symptoms	Emotional Hyper reactivity	Excessive Spending	Impaired social cognition	Affective blunting	Desinhibition	Social retreat	Apathy	Stereotypies	Number of treatments prior to diagnosis	number of treatments at last follow-up	AP efficacy	AP Intolerance
+	+	+	−	+	+	−	+	+	NA	0 (0)	0 (0)	NR	NR
+	+	+	+	+	+	+	−	−	NA	5 (2)	2 (0)	0	NA
+	+	+	−	NA	−	+	+	+	NA	3 (1)	2 (0)	1	NA
+	+	+	+	NA	NA	+	+	+	NA	13 (5)	5 (2)	0	NA
−	−	−	−	+	−	+	+	+	+	5 (2)	4 (2)	0	NA
+	NA	+	NA	NA	NA	NA	NA	+	NA	13 (4)	NA	0	NA
+	+	+	+	+	+	−	+	+	+	4 (2)	1 (1)	0	NA
−	−	−	+	+	+	+	+	+	+	8 (6)	2 (2)	0	0
+	−	−	+	+	−	+	+	+	+	9 (5)	NA	0	1
+	NA	+	−	+	−	+	+	+	NA	12 (6)	NA	0	NA
80 (8/10)	66 (6/9)	70 (7/10)	55 (5/9)	100 (7/7)	50 (4/8)	77 (7/9)	88 (8/9)	90 (9/10)	100 (4/4)	7,2 (3,3)	2,3 (1)	11 (1/9)	50 (1/2)

*Data for jealousy, prejudice, mystic and culpability NA

*Mgst* Miglsutat, *AP* Anti-psychotic

In the two first columns of the “psychotropic treatments”, the number in brackets refers specifically to anti-psychotic treatment (that are considered to be a sub-group of psychotropic treatments), whereas psychotropic treatments include also antidepressant, anxiolytics, and mood stabilizers (see Additional file 2: Table S2)

**Box 1:** Detailed history of three NP-C patients who suffered from psychotic episodes

Patient 5	A 25-year-old man was referred to our Neurology Unit presenting with a 9-year treatment-resistant schizophrenia, and experiencing recent gait disturbances. He was a single child. His parents reported a speech delay and learning difficulties in his childhood. He dropped out of school at the age of 15. He was described as introverted, usually playing alone, with restricted interests (with very specialized knowledge in insects and horses). At the age of 16, the patient showed behavioral changes. He described that “cyber-devils” were spying on him, which led him to jump out of a window from the first floor in order to escape them. He started to behave strangely, getting out of a supermarket with a shopping cart full of food he did not pay for, or suddenly getting naked in front of his parents at home without being able to explain his behavior. He also presented with social isolation, apathy, and anosognosia. The patient was hospitalized in a Psychiatric Unit, with a diagnosis of acute psychotic episode. He was treated with Risperidone 2 mg od, with partial efficacy. To be noted, his psychiatrist already reported a comorbid space and time disorientation and reversible acute faecal incontinence at this time. A second acute psychotic episode occurred at the age of 19, with similar psychotic symptoms, along with irritability, fluctuating space and time disorientation, hyperphagia, emotional blunting and discrete gait disturbances. A diagnosis of schizophrenia was eventually made. Various psychotropic drugs were prescribed (among which amisulpride, valproic acid, paroxetine, fluoxetine), alone or in association, without efficacy but with significant side effects such as extrapyramidal syndrome, dyskinesia and akathisia. It led to a switch for clozapine 200 mg od, with partial efficacy. A significant change in his behavior convinced his psychiatrist to refer him to a neurologist. He experienced stereotyped behaviors, endlessly repeating daily rituals (hairdressing, taking a shower several times a day, singing songs for hours for no reason, with uncontrollable laughter) and stereotyped speech (saying “that’s it” at the end of each sentence). His neurologist confirmed the presence of an associated cerebellar ataxia, with global slowness of movement, vertical supranuclear palsy, developmental delay and a more recent cognitive frontal syndrome. A diagnosis of Niemann-Pick type C disorder associated with autism spectrum disorder with psychotic features was eventually made with positive NP-C biomarkers (oxysterols) and genetic sequencing
Patient 8	A 25-year-old man was referred to our Neurology outpatient Clinics for exploration of a gait disturbance with cerebellar ataxia, dysarthria, dysphagia and behavioral changes. The patient reported a first psychotic episode at the age of 22, associated with a chronic history of cannabis abuse, leading to an admission in psychiatric wards. At this period of time, he expressed a polythematic delusion (with persecution, megalomania, mysticism, shouting “I am not a fundamentalist!” and autobiographic misconceptions “I am Bryan’s father, a sick son who was born premature”), auditory hallucinations, along with anosognosia. He was agitated, with disinhibition (dealing drugs in the hospital), apathy, impairment in social cognition, affective blunting, sleep disturbances and social retreat. Neurological symptoms were already identified, with cognitive impairment, dysarthria, dysphagia, gait disturbance and oculomotor dysfunction. He was first treated with various antipsychotics without clinical improvement until Clozapine 200 mg once daily (od) was introduced, allowing the patient to clinically improve. Her sister was diagnosed with paranoid schizophrenia. A diagnosis of Niemann-Pick type C disorder associated with a frontal lobe syndrome was eventually made after complementary investigations. Miglustat was introduced shortly after (200 mg three times per day), reducing drastically the course of the disease. The patient maintained a good level of autonomy through the years, with mild residual symptoms such as a dysexecutive syndrome, gait disturbance and impulsivity. He could walk without help. The patient discontinued the miglustat treatment after 3 years for a period of five months, leading to a worsening of the neurologic and psychiatric symptoms (acute psychotic relapse, severe dysphagia). A clinical improvement was reported after having reintroduced the Miglustat and Clozapine. After two years of psychotic remission, Clozapine was discontinued by his psychiatrist, due to a severe apathetic syndrome. Clinical remission was obtained for three more years, until an acute psychotic relapse was diagnosed, in a context of cannabis use. The patient described a persecutory delusion towards his parents, accusing them of stealing money from him, showing an aggressive behavior and elated mood. The patient was hospitalised and successfully treated with Risperidone 4 mg od and Quetiapine 200 mg od. Until now, the patient has been treated with Miglustat and antipsychotic medication
Patient 10	A 41-year-old woman was referred to our Neurology Unit with a 10-year treatment-resistant schizophrenia associated with recently screened cognitive impairment. She did not report any psychiatric episode until the age of 30. She was working in a supermarket, and lived with her husband and two children. Her sister was described as physically disabled since the age of 10 The patient experienced her first acute psychotic episode at the age of 30. At first, she reported insomnia, fatigue and loss of appetite leading to weight loss, for several days after having been a victim of a robbery at work. Then, the patient experienced behavioral changes. She became apathetic, crying every day. She told her husband that one of her colleagues was jealous of her, insulting her at work. She also believed her husband had poisoned their son, which led her to make her son vomit. She was convinced that her neighbors wanted to kill her and her family. Along with these delusions of persecution, she felt guilty at work. She feared being blamed for her incompetence at work, incompetence which was not confirmed by her staff. She did not criticize these ideas. She was first treated with haloperidol 10 mg od and cyamemazine 50 mg twice daily when admitted to the Psychiatric Unit During the next years, the patient was hospitalized 8 times, after having re-experienced psychotic symptoms of persecution associated with auditory hallucinations. A diagnosis of schizoaffective disorder was made. Various psychotropic drugs were prescribed (among which Amisulpride, Chlorpromazine, Valproic Acid, Fluoxetine, Clomipramine), alone or in association, without efficacy for more than two months. At the age of 40, the patient discontinued her treatment, leading to a relapse of an acute psychotic episode. She was first treated with Olanzapine 10 mg od. This treatment led to numerous side effects, including somnolence and acute delirium. A switch for Risperidone 1 mg od was eventually decided. At this time, one of the psychiatrists screened the patient for cognitive impairment. It revealed a dysexecutive and attentional syndrome, along with apraxia. A Brain Magnetic Resonance Imaging (MRI) was performed, revealing a right frontal lobe atrophy. She was then referred to a neurologist, who found a cerebellar ataxia and a vertical supranuclear palsy. The cognitive impairment was confirmed by the neuropsychological assessment. A diagnosis of Niemann Pick type C disease with psychotic features was eventually made after a positive filipin test on a cutaneous biopsy and genetic sequencing

**Fig. 1:**
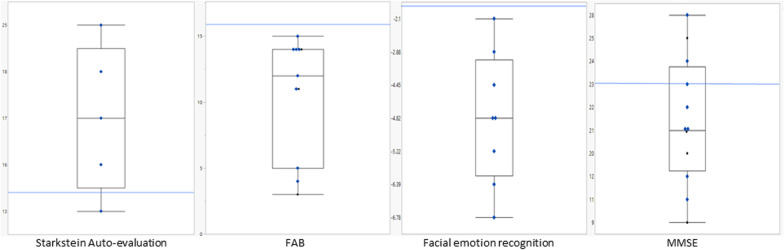
Scores of individuals NP-C patients for Starkstein auto-evaluation scales, FAB (Frontal Assessment Battery), FER (Facial Emotion Recognition), and MMSE (Mini Mental State Examination), displayed as box plots indicating median, interquartile range, and extreme values for each test. The blue line indicates the normal value limit (normal below the blue line for Starkstein, above for FAB, above for FER, and above for MMSE)

Beyond psychosis, most of the patients suffered from behavioral (15/17) and cognitive disorders (18/19). The only patient without a cognitive complaint (whereas not formally tested) was one out of the two without any behavioral disorder detected. MMSE [10] mean score was 19.75 (SD ± 5.93, 9–28, n = 12), whereas FAB [11] mean score was 10.64 (SD ± 4.48, 3–15, n = 11) (see Fig. 1). Executive functions (based on frontal lobe functioning) were altered in 11/12 patients tested. Numerous frontal abnormal behaviors were observed in our cohort: apathy (13/17), impaired social cognition (11/13) and stereotyped behaviors (5/10). For five patients (patients 4, 8, 12, 15 and 16) quality of life was markedly impaired by these abnormal behaviors, being the principal burden of the disease, as detailed for patient 15 (see Box 2). As for psychometric and neuropsychological tests evaluating social cognition underlying frontal behavioral disorders, facial emotion recognition test (FER) [14] was by far the most frequently performed (n = 8), as FER require minimal cognitive skills. FER scores were abnormal in all of tested patients (see Fig. 1).

**Box 2:** detailed history of one NP-C patient whose main disability was related to a frontal behavior manifestations

Patient 15	A 33-year-old man of Moroccan origin was referred to our Neurology outpatient Clinics for exploration of behavioral changes, along with a 15 year-long learning disability and more recent gait disturbances. The patient and his family reported no history of psychiatric/neurologic disorder. He was born from consanguineous parents (cousins’ siblings), at term, without jaundice. He started to experience learning difficulties (mathematics) at the age of 18, repeating twice his last grade at high school. He could not properly work and stayed at his parents’ house. He did not invest his social life. His parents described him as more and more introverted since the age of 28. He started to experience gait disturbances at the age of 30. His family reported insidious behavioral changes including apathy (spending the day watching TV) and personal neglect. He bought expensive pairs of shoes, mobile phones, without elaborating more about it. He withdrew large amounts of cash several times and started to answer to misleading advertising asking for money. The patient acted like he was not aware of this behavioral change. Apart from that, the patient did not report any delusion/hallucination, depressive symptoms or anxiety. After a neurological assessment, we suspected a cognitive and behavioral complication of a frontal lobe syndrome, along with learning disability, cerebellar ataxia and vertical supranuclear palsy. A brain Magnetic Resonance Imaging revealed a bifrontal atrophy. Neuropsychological assessment confirmed a dysexecutive syndrome and an attention deficit. A diagnosis of Niemann-Pick type C disorder associated with a frontal lobe syndrome was eventually made with positive NP-C biomarkers (oxysterols) and genetic sequencing

## Discussion

We report a high frequency of psychiatric symptoms in NP-C encompassing acute psychotic manifestations, presenting often early in the course of the disease with atypical features, and disabling behavioral manifestations mostly due to frontal dysfunction.

Psychotic disorders are frequent in the general population, with a pool incidence of 26.6 per 100,000 person-years [18]. More interestingly, prevalence of Clinical High-Risk for Psychosis (defined by the presence of attenuated psychotic symptoms, functional impairment for more than a year and an accumulation of numerous risk factors for psychosis in a patient) is estimated at 1.7% in the general population and 19.2% in clinical samples [32]. Psychosis in childhood and in young adults is mostly related to paranoid schizophrenia but may sometimes be part of an inborn error of metabolism, rare genetic diseases that in some cases may have disease-modifying therapies, such as in NP-C [6–8, 13, 15],Psychotic symptoms in NP-C patients has been reported in up to 55% of patients [23]. Thus, diagnosing NP-C disease in patients presenting with an acute psychotic episode is critical but challenging for psychiatrists.

This series of NP-C patients aimed to reveal some atypical clinical features that should lead to blood biomarker screening for NP-C disease in patients with an acute psychotic episode: i) history of psychomotor delay and/or autistic symptoms before the onset of psychosis; ii) associated neurological symptoms (including delirium, cognitive symptoms and frontal behavioral symptoms); iii) complex delusion, defined as a delusion including multiple themes concomitantly; iv) exclusive visual hallucinations and v) antipsychotic lack of efficiency and/or intolerance.

Concerning childhood reported manifestations, there is growing evidence of an overlap between autistic spectrum disorder and schizophrenia [4, 19, 34], and individuals at ultra-high risk for psychosis may present mild psychiatric or neurological symptoms during the prodromal phase such as thoughts, language, perception and motor disturbances, disorders of emotion and affect or impaired social ability [1, 2, 33]. Nevertheless, these prodromal neurodevelopmental symptoms remain relatively mild and do not impair psychomotor development until late teenage years and young adulthood in patients with classical paranoid schizophrenia, which is not the case in NP-C patients with psychosis. In addition, schizophrenic patients may present with neurological soft signs, ie subtle deficits in sensory integration, motor coordination, and sequencing of complex motor acts altered. NP-C patients also suffer from motor manifestations, mainly cerebellar ataxia and dystonia (that may be thought to be of iatrogenic origin), which are more prominent and disabling than the so-called neurological “soft signs”. A characteristic manifestation of NP-C is Vertical supranuclear gaze palsy (VSGP), that was present in all NP-C patients in this series. Unfortunately, this data was almost never available at first presentation, but as VSGP is described as an early manifestation of NP-C often without complaint, we consider that VSGP should be specifically looked for in evaluating psychotic patients, especially when atypical signs are described.

Considering the nature of the psychotic symptoms, although delusions (especially persecutory) are a hallmark of schizophrenia and one of the main diagnostic criteria for the disorder [3, 21], complex delusions are rather rare. Nonetheless, it was a frequent clinical feature reported in our patients. Exclusive visual hallucinations are classically highly suggestive of an associated organic disease, since visual hallucinations in schizophrenia are mostly complex and co-occur with auditory hallucinations [35]. Interestingly, patients did not report being aware of the hallucination process, similarly to schizophrenic patients.

Furthermore, almost all of our patients reported treatment resistance (antipsychotic lack of efficiency) and/or treatment intolerance referred an atypical clinical feature in NP-C, but only in retrospective studies [31].

Little is known about the underlying mechanisms involved in such resistance. One hypothesis may be that the defect in transporting cholesterol and other fatty substances (lipids) inside of cells as seen in NP-C (leading to an abnormal accumulation of cholesterol) may play a role in AP efficacy. Some recent studies have raised that a change in polyinsaturated fatty acid levels may be associated with diminished AP treatment response in chronic psychosis [20]. Such association may explain why NP-C patients commonly report AP resistance.

In our NP-C patients, cognitive disorder was common (18/19) and in eight patients, extensive neuropsychological evaluation was performed, where at least one abnormal cognitive test pointing out frontal dysfunction was present in all eight patients. Although this has already been described by Heitz et al. [17], we present additional data regarding behavioral manifestations, that were present in 15/17 patients. Interestingly, the main processes involved specifically in social cognition (facial emotion recognition and Theory of Mind) were altered in all patients who underwent the tests. Considered together, one may argue that frontal lobe dysfunction may participate in the development of behavioral symptoms in addition to cognitive impairment in NP-C patients, since social cognition impairment is related to orbitofrontal dysfunction [16, 28]. Moreover, cognitive impairment may contribute to the occurrence of psychotic symptoms, through the combination of two cognitive bias: the “jumping to conclusions” bias (tendency to make rushed and premature decisions on the basis of little evidence) and the “evidence integration” bias (impaired integration of disambiguating evidence) [24].

Cognitive/behavioral impairment in NP-C may be due to disruption in brain development, neurodegeneration, or a combination of both. Neurodevelopmental disorders are often associated with psychiatric features (psychotic and/or autistic symptoms) and have a major impact on cognition as the frontal lobe is developing until adulthood. Social cognition impairment is common to psychosis and autistic spectrum disorder [16, 28]. Neurodegeneration with specific involvement of fronto-striatal circuits may also contribute to frontal lobe-predominant cognitive/behavioral impairment in NP-C patients, as some clinical characteristics (motor manifestations, executive dysfunction, apathy) may resemble Frontotemporal Dementia (FTD) [5, 12, 25, 36]. No correlation can be made in our study in terms of the specific brain area involved, but further studies may help correlate MRI abnormalities and psychometric tests.

Miglustat has a positive impact on global cognition [17], and thus could be of great impact on frontal lobe dysfunction and its underlying behavioral disorders, at least to avoid progressive worsening. We did not have access to longitudinal specific psychometric scales to observe evolution of behavioral disorders. On the other hand, only 2/7 patients had a psychotic relapse under Miglustat treatment, and one of them (patient 8) occurred after rapid reduction of anti-psychotics and cannabis use. Therefore, miglustat seemed to prevent psychotic relapse, and this may could be partly explained by its effect on cognition.

## Conclusion

Psychiatric symptoms in NP-C disease are common and can frequently appear in the form of an acute psychotic episode. Associated atypical signs, such as a history of psychomotor delay and/or autistic symptoms before the onset of psychosis, complex delusions, isolated visual hallucinations, treatment resistance or intolerance, and/or neurological symptoms may prompt NP-C screening to permit the early initiation of appropriate treatment, and may protentially prevent psychotic relapses. Behavioral manifestations in the form of a frontal syndrome may have a higher burden on quality of life that classically described motor and cognitive symptoms in NP-C patients.

### Supplementary Information

**Additional file 1**. **Table S1**: exhaustive clinical and paraclinical data of the 19 included NP-C patients.

**Additional file 2**.** Table S2**: psychotropic treatments include also antidepressant, anxiolytics, and mood stabilizers of the 10 ncluded NP-C-psychosis patients.

## Data Availability

The datasets used and/or analysed during the current study are available publicly: https://dispose.aphp.fr/u/yAgOk2OIDqLaCA6V/c49bb020-1479-4e12-9e43-8a218bd17902?l
